# Supplementary material for: MHASS: Microbiome HiFi Amplicon Sequencing Simulator
Source: Bioinformatics. 2025 Dec 6;42(1):btaf656. doi: 10.1093/bioinformatics/btaf656 (PMC12790812; doi:10.1093/bioinformatics/btaf656)
Supplement: btaf656_Supplementary_Data [file btaf656_supplementary_data.pdf]

Supplementary Figures

Please note that AmpliconHunter requires the user to have a local copy of all genomes from which amplicons should be extracted. As this may overwhelm some environments, we also supply a webserver at `https://ah1.engr.uconn.edu` to allow users to obtain such amplicons from large precompiled genomic databases, without needing to store any intermediate files locally.

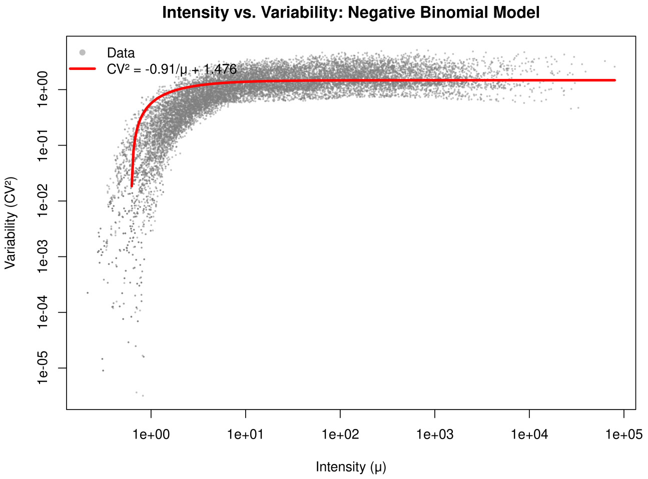

Fig. S1. Variability model fitting for abundance simulation in MHASS. To determine appropriate variability parameters for metaSPARSim, we analyzed the relationship between intensity μ (mean abundance) and variability (CV^2) using the R1 dataset provided by metaSPARSim. The data exhibited a negative binomial-like relationship where $CV^2 = \beta_1/\mu + \beta_0$. Linear regression on CV^2 vs. $1/\mu$ yielded $CV^2 = -0.910/\mu + 1.476$ with $R^2 = 0.370$. As mean abundance increases, the coefficient of variation decreases, consistent with overdispersed count data. The negative coefficient for the $1/\mu$ term (-0.910) deviates from the theoretical value of 1.0 for a true negative binomial distribution, suggesting the microbiome abundance data follows a modified overdispersion pattern. Data points are shown in gray, the fitted regression line in red, and axes are log-transformed.

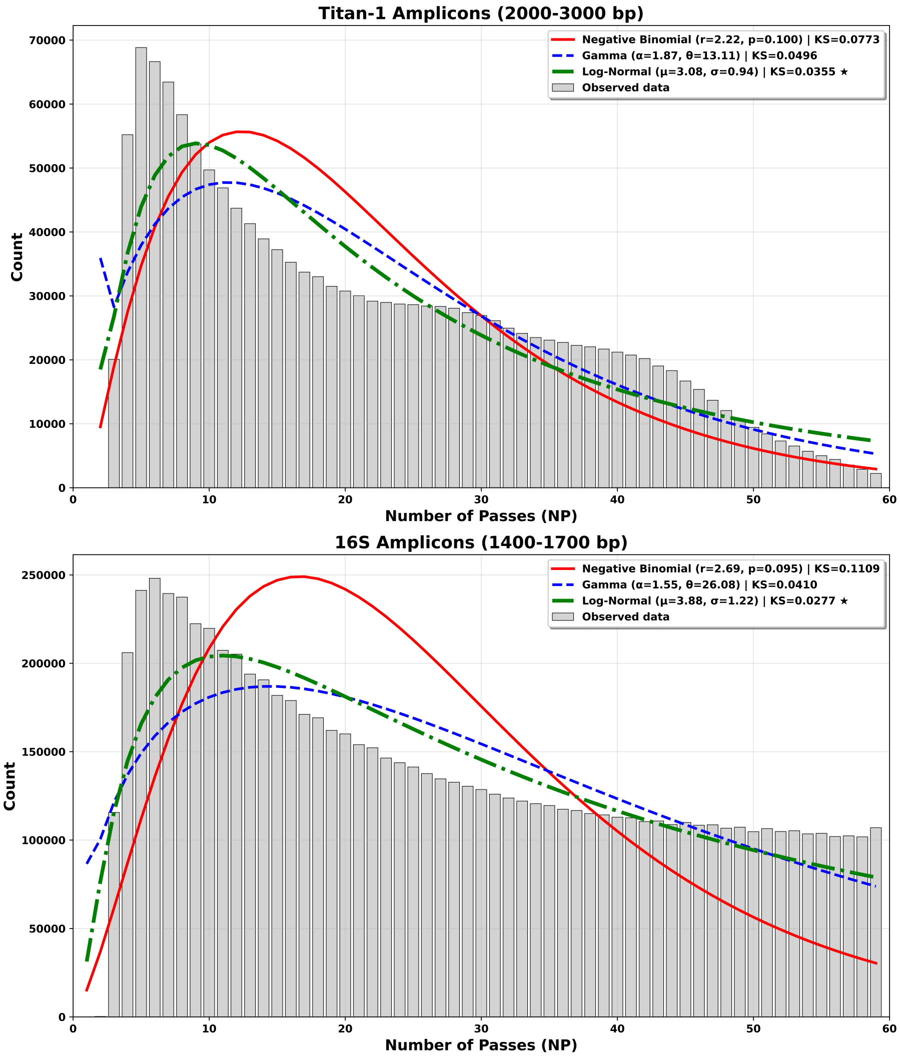

Fig. S2. Number of passes from two datasets with CCS annotations are fit to three models. The top panel represents the distribution of number of passes for Titan-1 amplicons (Howard-Stone et al., 2025), reads filtered to a length of 2,000-3,000 bp. The bottom panel represents the same distribution for a dataset of 16S amplicons `https://downloads.pacbcloud.com/public/dataset/Kinnex-16S/DATA-ATCC-MSA1003-96plex-Revio-Monomer/`, filtered to 1400-1700 bp in length. Three models are fit to each distribution, with Log-Normal obtaining the lowest Kolmogorov–Smirnov statistic for both datasets.

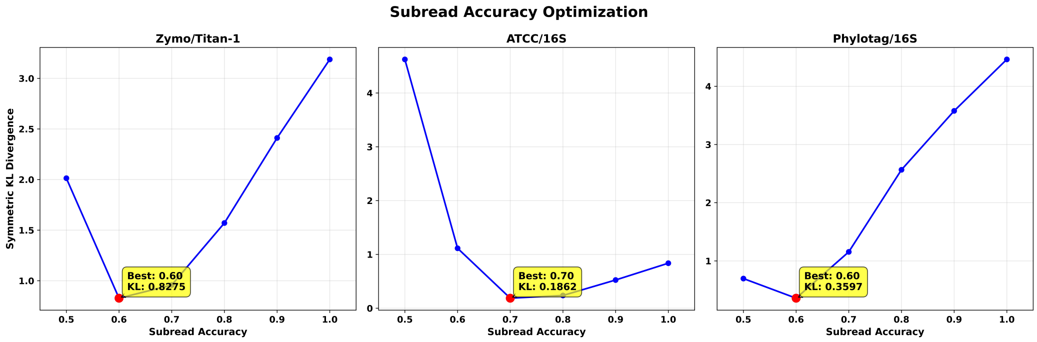

Fig. S3. Subread accuracy optimization results for all three datasets (from left to right): Zymo/Titan-1, ATCC/16S, and Phylotag/16S. The subread accuracy parameter for PBSIM3 was optimized independently for each dataset by testing accuracy values from 0.5 to 1.0 in increments of 0.1, and running complete simulations for each value. The optimal accuracy was selected by minimizing the Kullback-Leibler (KL) divergence between the edit distance distributions of real and simulated reads. KL divergence between the distributions of minimum edit distances from reads to references for real vs. simulated reads are shown in blue. The optimal values are highlighted in red with and annotated with their KL divergence values and optimal subread accuracy: 0.6 for Zymo/Titan-1, 0.7 for ATCC/16S, and 0.6 for Phylotag/16S.

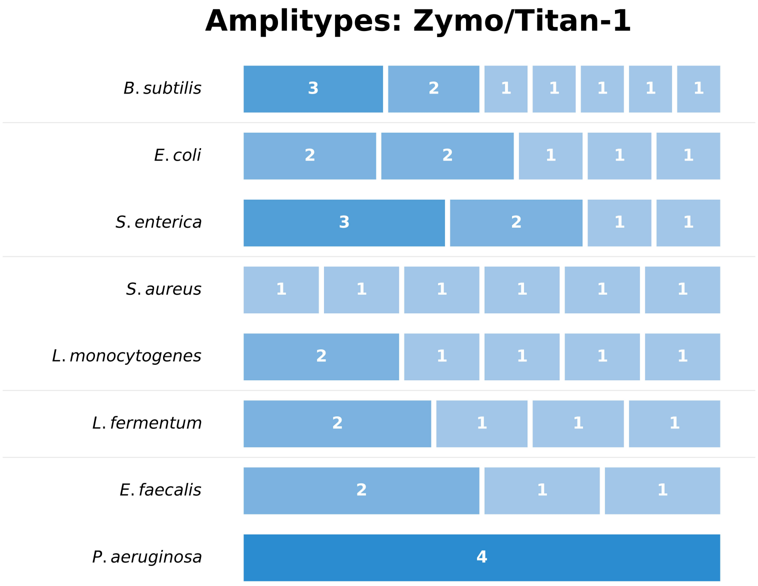

Fig. S4. Amplicon multiplicity types detected in the Zymo/Titan-1 dataset, showing the multiple amplicon variants per genome with copy number.

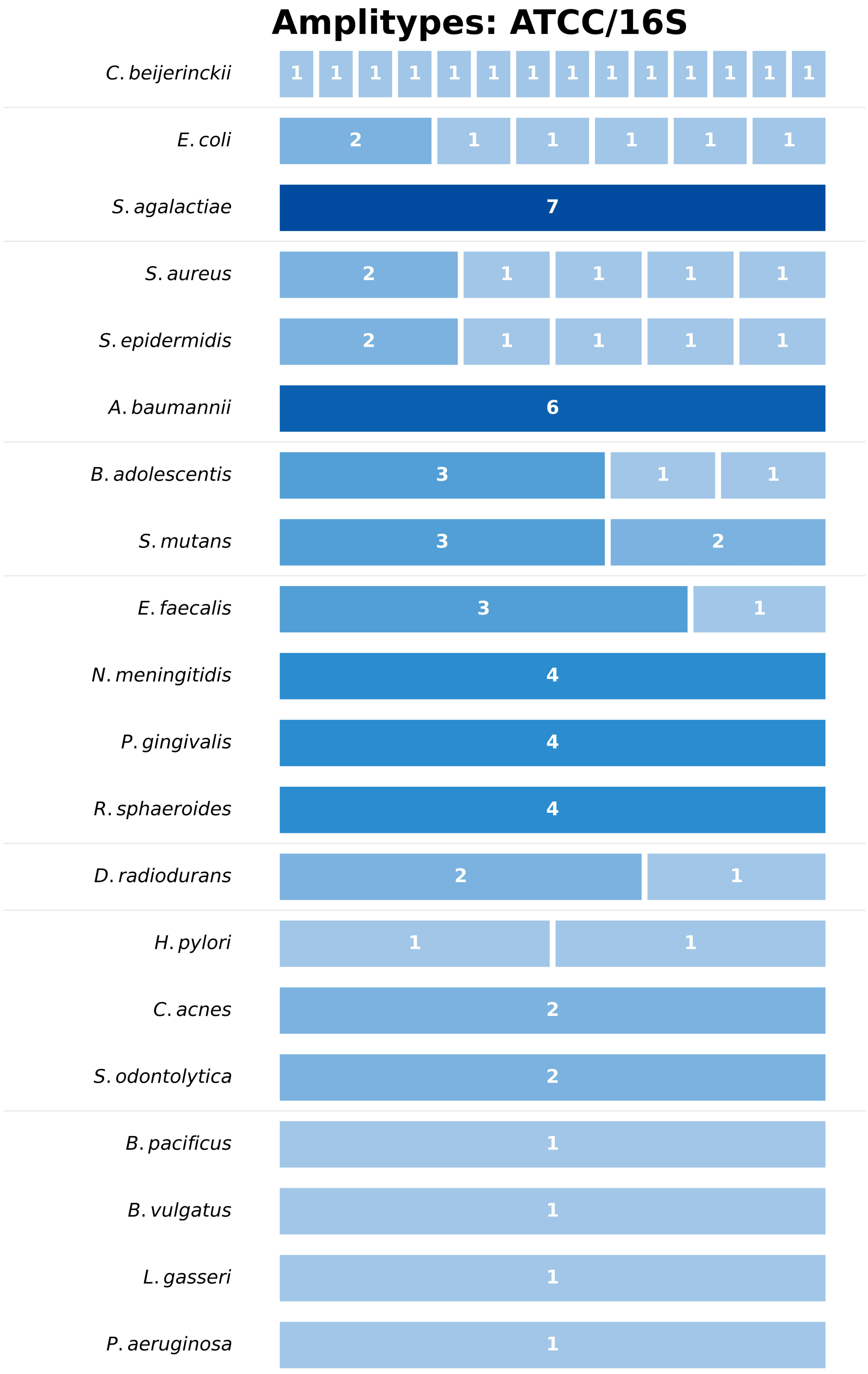

Fig. S5. Amplicon multiplicity types detected in the ATCC/16S dataset, showing the multiple amplicon variants per genome with copy number.

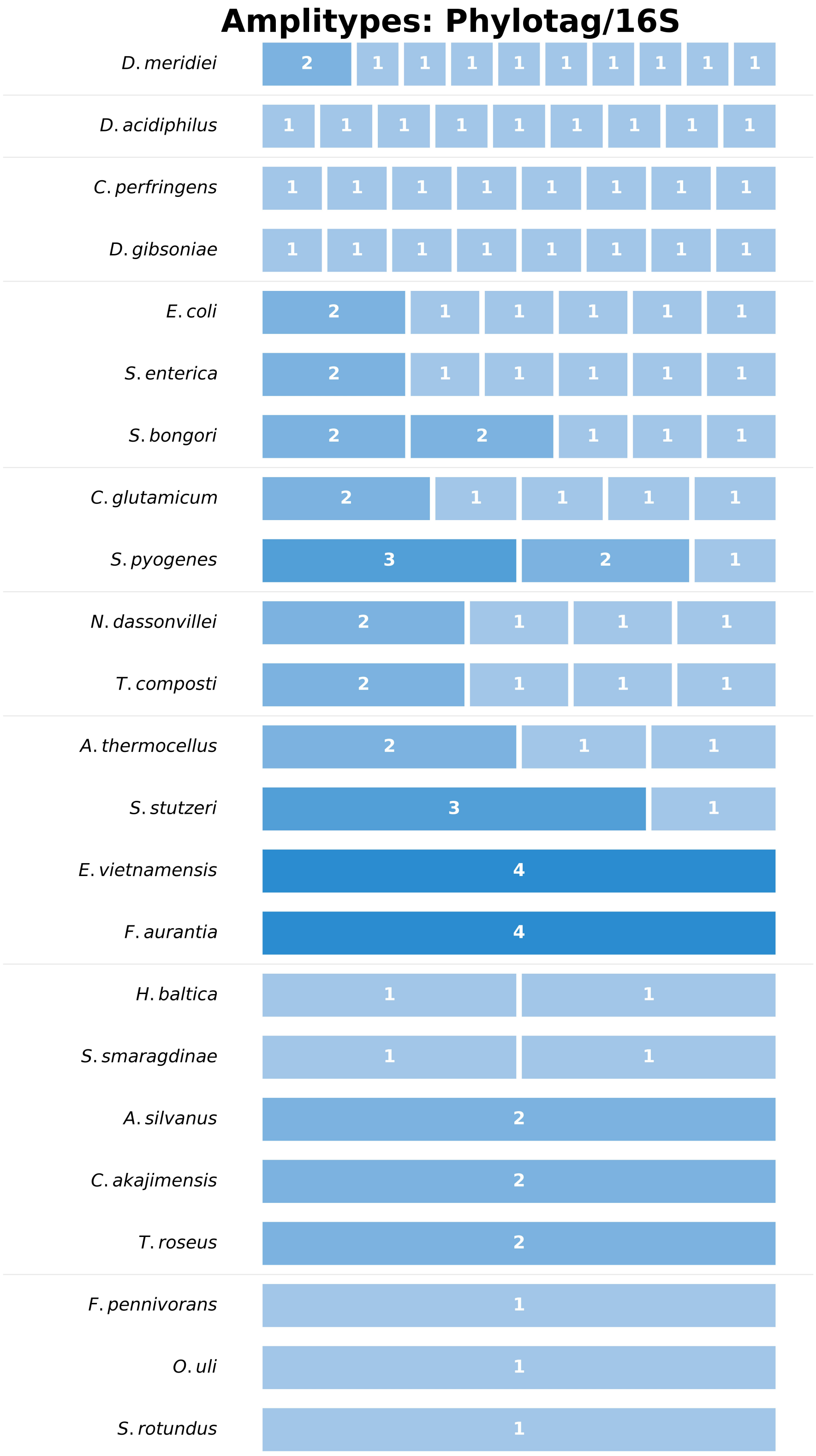

Fig. S6. Amplicon multiplicity types detected in the Phylotag/16S dataset, showing the multiple amplicon variants per genome with copy number.

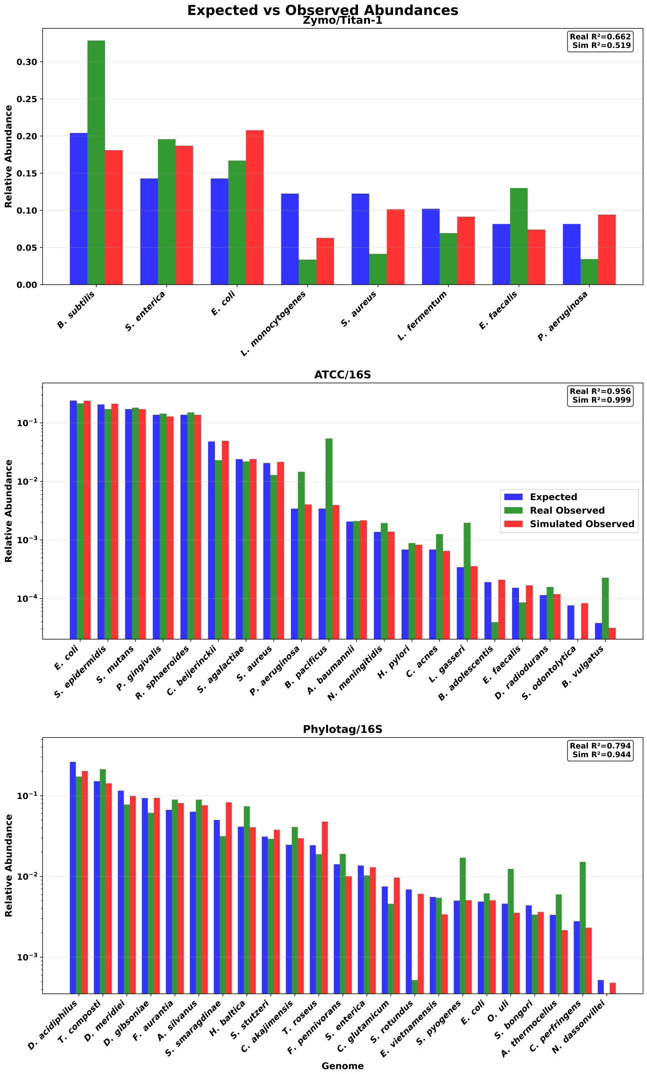

Fig. S7. Bar plots showing expected (based on known mock community composition) versus observed ASV abundances at the genome level for both real and simulated data. Correlation coefficients (R^2) demonstrate high fidelity of abundance simulation across all three datasets. Abundances for the Zymo/Titan-1 and ATCC/16S datasets are known and published in their respective product sheets (Zymo DS1706, and ATCC MSA-1003, respectively). The custom mock community, Phylotag/16S, was produced by the authors of Singer et al. (2016), who note pipetting errors in mixing. To circumvent this, we used their measurements of short read shotgun data as the source of truth for genome abundance, rather than molarity, as the authors suggest. Values were estimated from Supplementary Figure 4 of Singer et al. (2016), using `https://www.graphreader.com/` Larsen (n.d.).

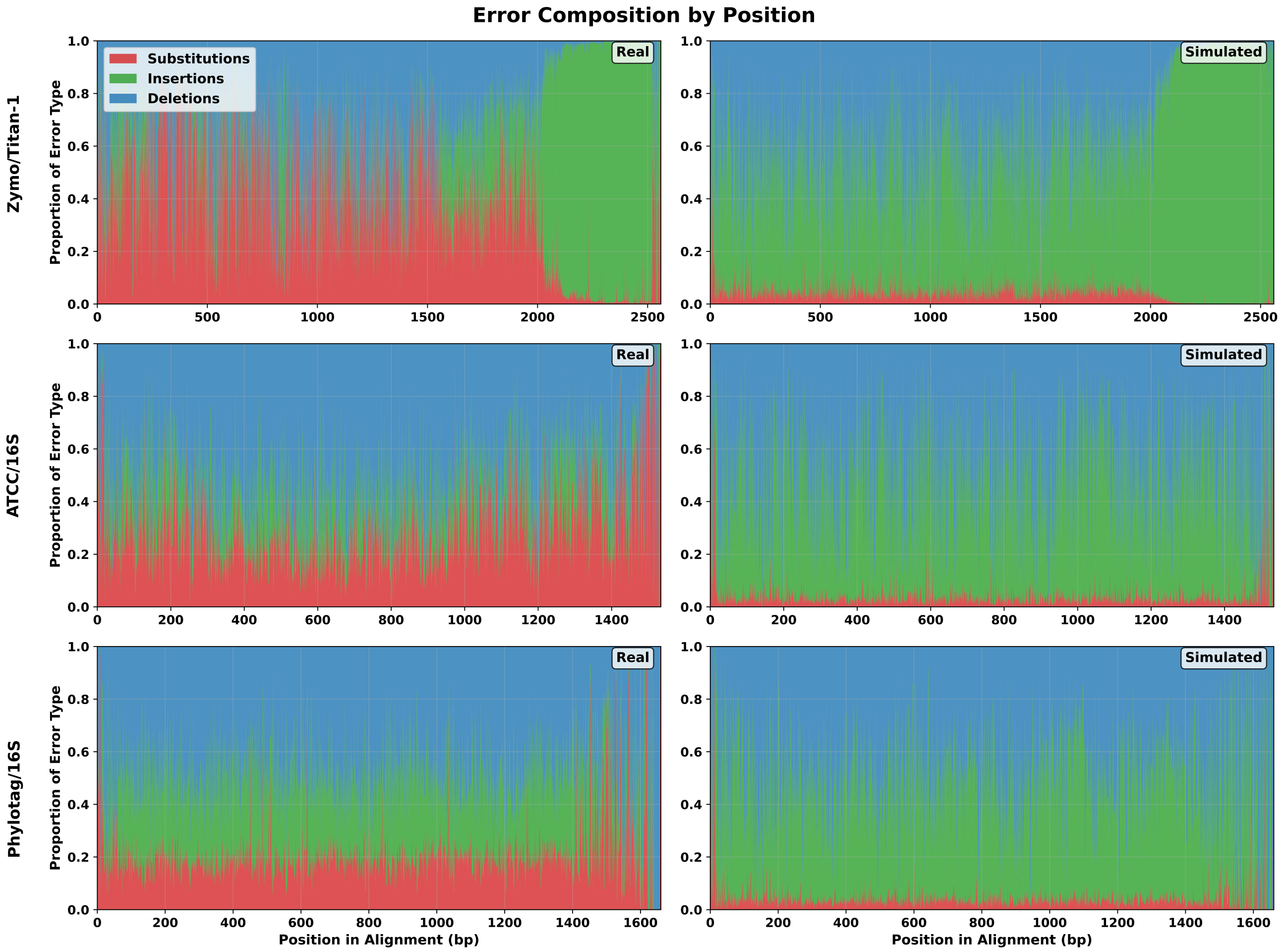

Fig. S8. Each read is aligned globally to its nearest reference ASV. Proportion of errors by type and position in alignment is shown for all three datasets (from top to bottom): Zymo/Titan-1, ATCC/16S, and Phylotag/16S. Substitutions are shown in red, insertions in green, and deletions in blue. Real data is shown on the left, simulated data is shown on the right. A notable increase of insertions is seen in both real and simulated reads for the Zymo/Titan-1 dataset from approximately position 2100 to the end of the alignment. The proportion of substitutions in real data is much higher than would be implied by the default error ratio parameter supplied by PBSIM3 for the RSII (Ono et al., 2022).

Supplementary Tables

Table S1. Feature comparison of microbiome simulation tools

Feature	metaSPARSim	PBSIM3	CAMISIM	miaSim	MHASS
Abundance	✓		✓	✓	✓
Copy #					✓
Barcoding					✓
Sequencing errors		✓	✓		✓
CCS-compatible	✓	✓		✓	✓
Pass number distribution					✓

Table S2. Dataset information and computational resource usage for MHASS evaluation. Performance was measured on a virtual machine configured with 180 virtual cores and 374GB RAM running on a Dell PowerEdge R7525 server with two AMD EPYC 7552 48-core CPUs. CPU usage indicates effective parallelization across multiple cores, with an average core usage of 100/180. Runtime includes all pipeline steps starting with ASV extraction from genomes, through to final FASTQ generation.

Dataset	Platform	Total Reads	Samples	Runtime (h:mm:ss)	Peak Memory (GB)
Zymo/Titan-1	Sequel II	186,167	96	0:40:23	1.8
ATCC/16S	Sequel II	2,468,174	192	6:45:55	8.8
Phylotag/16S	RSII	113,709	5	0:22:36	0.9

Table S3. Dataset Information - Zymo/Titan-1 (D6300)

Species	Abundance (%)	Copy Number
Bacillus subtilis	12.5	10
Enterococcus faecalis	12.5	4
Escherichia coli	12.5	7
Lactobacillus fermentum	12.5	5
Listeria monocytogenes	12.5	6
Pseudomonas aeruginosa	12.5	4
Salmonella enterica	12.5	7
Staphylococcus aureus	12.5	6

References

R. Howard-Stone, P. Gerwin, D. Capunitan, et al. Cecal microbiome transplantation without antibiotic preconditioning standardizes murine microbiomes. *Frontiers in Microbiology*, 16, 2025. doi: 10.3389/fmicb.2025.1632210.

K. Larsen. Online tool for reading graph image values. `https://www.graphreader.com/`, n.d. Accessed: May 27, 2025.

Y. Ono, M. Hamada, and K. Asai. PBSIM3: a simulator for all types of pacbio and ont long reads. *NAR Genomics and Bioinformatics*, 4(4):lqac092, 2022. doi: 10.1093/nargab/lqac092.

E. Singer, B. Bushnell, D. Coleman-Derr, et al. High-resolution phylogenetic microbial community profiling. *The ISME Journal*, 10:2020–2032, 2016. doi: 10.1038/ismej.2015.249.

Table S4. Dataset Information - ATCC/16S (MSA-1003)

Species	Accession	Abundance (%)	Copy Number
Acinetobacter baumannii (ATCC 17978)	GCF_902728005.1	0.18	6
Bacillus pacificus (ATCC 10987)	GCF_031316815.1	1.8	1
Bacteroides vulgatus (ATCC 8482)	GCF_028538915.1	0.02	1
Bifidobacterium adolescentis (ATCC 15703)	GCF_000010425.1	0.02	5
Clostridium beijerinckii (ATCC 35702)	GCF_000767745.1	1.8	14
Cutibacterium acnes (ATCC 11828)	GCF_000231215.1	0.18	2
Deinococcus radiodurans (ATCC BAA-816)	GCF_000008565.1	0.02	3
Enterococcus faecalis (ATCC 47077)	GCF_004006275.1	0.02	4
Escherichia coli (ATCC 700926)	GCF_000364365.1	18	7
Helicobacter pylori (ATCC 700392)	GCF_000008525.1	0.18	2
Lactobacillus gasseri (ATCC 33323)	GCF_008868295.1	0.18	1
Neisseria meningitidis (ATCC BAA-335)	GCF_000008805.1	0.18	4
Porphyromonas gingivalis (ATCC 33277)	GCF_002892575.1	18	4
Pseudomonas aeruginosa (ATCC 9027)	GCF_001294675.1	1.8	1
Rhodobacter sphaeroides (ATCC 17029)	GCF_000015985.1	18	4
Schaalia odontolytica (ATCC 17982)	GCF_000154225.1	0.02	2
Staphylococcus aureus (ATCC BAA-1556)	GCF_032809245.1	1.8	6
Staphylococcus epidermidis (ATCC 12228)	GCF_022869565.1	18	6
Streptococcus agalactiae (ATCC BAA-611)	GCF_000007265.1	1.8	7
Streptococcus mutans (ATCC 700610)	GCF_000007465.2	18	5

Table S5. Dataset Information - Phylotag/16S

Species	Accession	Abundance (%)	Copy Number
Acetivibrio thermocellus	GCF_000015865.1	0.3451	4
Allomeiothermus silvanus	GCF_000092125.1	13.11167	2
Clostridium perfringens	GCF_000013285.1	0.14404	8
Coraliomargarita akajimensis	GCF_000025905.1	5.09543	2
Corynebacterium glutamicum	GCF_000011325.1	0.51815	6
Desulfoscipio gibsoniae	GCF_000233715.2	4.83635	8
Desulfosporosinus acidiphilus	GCF_000255115.2	12.08939	9
Desulfosporosinus meridiei	GCF_000231385.2	4.34622	11
Echinicola vietnamensis	GCF_000325705.1	0.57616	4
Escherichia coli	GCF_000005845.2	0.28808	7
Fervidobacterium pennivorans	GCF_000235405.2	5.84364	1
Frateuria aurantia	GCF_000242255.2	6.90793	4
Hirschia baltica	GCF_000023785.1	8.54939	2
Nocardiopsis dassonvillei	GCF_000092985.1	0.04301	5
Olsenella uli	GCF_000143845.1	1.89953	1
Salmonella bongori	GCF_000252995.1	0.25907	7
Salmonella enterica	GCF_000018625.1	0.80623	7
Sediminispirochaeta smaragdinae	GCF_000143985.1	10.3629	2
Segniliparus rotundus	GCF_000092825.1	2.8498	1
Streptococcus pyogenes	GCF_000006785.2	0.3451	6
Stutzerimonas stutzeri	GCF_000327065.1	3.2239	4
Terriglobus roseus	GCF_000265425.1	5.03741	2
Thermobacillus composti	GCF_000227705.2	12.5215	5
